# Targeting neuronal epigenomes for brain rejuvenation

**DOI:** 10.1038/s44318-024-00148-8

**Published:** 2024-07-15

**Authors:** Sara Zocher

**Affiliations:** German Center for Neurodegenerative Diseases, Tatzberg 41, 01307 Dresden, Germany

**Keywords:** Neuron Aging, Cognitive Decline, Neuronal Epigenome, Epigenetic Rejuvenation, Epigenome Editing, Chromatin, Transcription & Genomics, Molecular Biology of Disease, Neuroscience

## Abstract

Aging is associated with a progressive decline of brain function, and the underlying causes and possible interventions to prevent this cognitive decline have been the focus of intense investigation. The maintenance of neuronal function over the lifespan requires proper epigenetic regulation, and accumulating evidence suggests that the deterioration of the neuronal epigenetic landscape contributes to brain dysfunction during aging. Epigenetic aging of neurons may, however, be malleable. Recent reports have shown age-related epigenetic changes in neurons to be reversible and targetable by rejuvenation strategies that can restore brain function during aging. This review discusses the current evidence that identifies neuronal epigenetic aging as a driver of cognitive decline and a promising target of brain rejuvenation strategies, and it highlights potential approaches for the specific manipulation of the aging neuronal epigenome to restore a youthful epigenetic state in the brain.

## Abstract (web summary)

This review discusses neuronal epigenetic aging as a driver of cognitive decline with age and highlights potential interventions for targeted brain rejuvenation.

## Graphical abstract

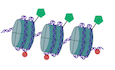

## Introduction

Aging is characterized by progressive impairments in brain function, which manifest in behavioral alterations and cognitive decline, as well as increased risk for neurodegenerative diseases (Hou et al, 2019). Despite significant research efforts in the last decades, mediators of brain aging that could be targeted to prevent or counteract brain aging are still largely unknown. Neurons undergo pronounced alterations in morphology and function throughout the lifespan, and these have been related to disturbed neuronal signaling and impaired information processing in the aged brain (Radulescu et al, 2021; Wang et al, 2011; Mattson and Arumugam, 2018). The function of different neuron types in multiple brain areas is affected by aging, with the hippocampus and prefrontal cortex—both brain regions with key roles in memory storage and cognitive flexibility—being particularly compromised (Morrison and Baxter, 2012; Fan et al, 2017). Since neurons are post-mitotic and mostly generated during early development, they represent one of the oldest cell types in the body. Therefore, to preserve their function throughout life, neurons are dependent on the long-term maintenance of molecular programs that define their neuronal identity and enable activity-induced plasticity in response to environmental cues. Yet, multiple studies have reported impairments in neuron-specific gene expression programs in the aging brain, including alterations in transcription, RNA processing, and protein levels, which have been linked to neuronal dysfunction (Hajdarovic et al, 2022; Stilling et al, 2014; Wingo et al, 2019; Lu et al, 2004; Zhang et al, 2022). The long-term maintenance of neuronal gene expression programs critically depends on the epigenetic machinery, and accumulating evidence suggests impairments of epigenetic regulation as cell-intrinsic drivers of aging in neurons.

The neuronal epigenome has been comprehensively characterized over the last two decades, and cumulative research has established its critical importance for synaptic plasticity, behavior, and cognition (Levenson et al, 2004; Alarcón et al, 2004; Korzus et al, 2004; Miller and Sweatt, 2007; Rudenko et al, 2013; Feng et al, 2010; Morris et al, 2014; Campbell and Wood, 2019; Coda and Gräff, 2024). Neurons have distinctive epigenetic patterns that differ from those of other cell types, and even neuron types and subtypes can be distinguished based on their unique epigenomes (Luo et al, 2017; Yao et al, 2021; Zhu et al, 2021; Liu et al, 2021). The proper establishment of neuron-specific epigenetic patterns during neuronal differentiation and neuronal maturation is critical for the development of neuronal gene expression programs and neuronal identities as well as for the functional maturation of neurons (Ziller et al, 2018; Zocher et al, 2021a; Gallegos et al, 2018). Once matured, neurons must maintain an intact epigenetic machinery long-term in order to preserve their function, with perturbations of this machinery leading to brain dysfunction (Oliveira et al, 2012; Von Schimmelmann et al, 2016; Lipinski et al, 2020; Kupke et al, 2024). The extensive changes of the neuronal epigenetic landscape that have been observed during aging imply that the ability of neurons to maintain their epigenetic profiles is impaired during aging (Peleg et al, 2010; Benito et al, 2015; Zocher et al, 2021b; Zhang et al, 2022, 2023). Intriguingly, recent studies suggest that neuronal epigenetic aging can be slowed down or reversed by rejuvenating interventions that are known to counteract age-related impairments in brain function (Hadad et al, 2018; Lu et al, 2020a; Zocher et al, 2021b), thus opening the field for therapeutic anti-aging strategies. Interventions shown to be effective include changes to lifestyle (such as exercise, environmental enrichment, and caloric restriction), the transfer of young blood factors or cellular reprogramming, among others (Benayoun et al, 2015; Zhang et al, 2020). The malleability of the neuronal epigenome during aging suggests that it can be targeted to prevent epigenetic aging or even restore a youthful epigenetic state in aged neurons.

This review investigates the role of neuronal epigenomes as molecular mediators of brain aging and potential targets to prevent cognitive decline. It first discusses the current evidence for neuronal epigenetic aging as a driver of cognitive decline, examining in detail different epigenetic modifications and highlighting current knowledge gaps and future research avenues. There follows a summary of recent studies that suggest neuronal epigenomes as critical targets and potential mediators of brain rejuvenation strategies. Finally, this article discusses approaches to specifically manipulate the aging neuronal epigenome with the goal to restore neuronal function in the aged brain.

## Epigenetic changes underlying neuron aging

**Figure 1:**
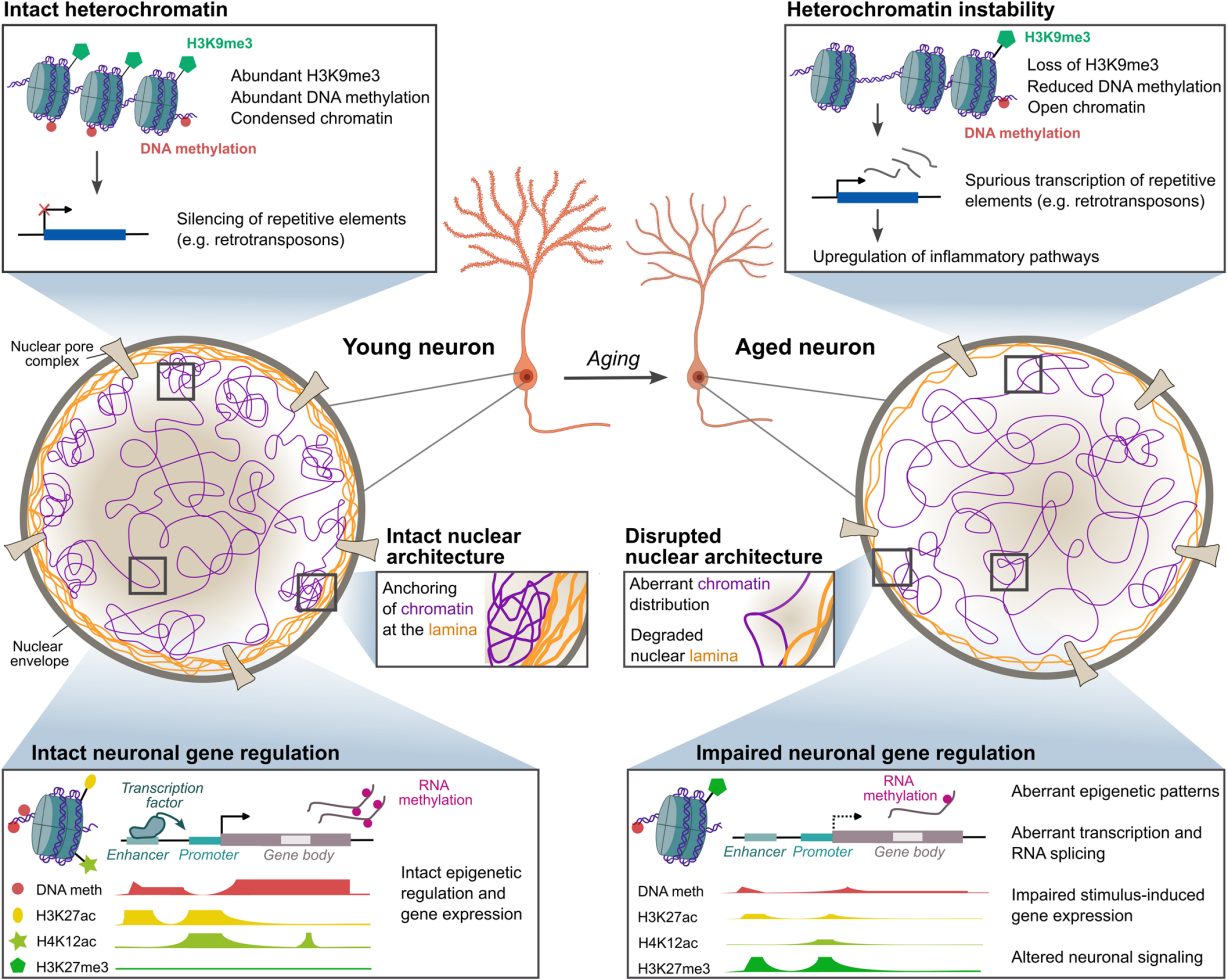
The aging neuronal epigenome. Neuron-specific epigenetic patterns are profoundly altered in the aged brain, contributing to impaired gene regulation and ultimately neuron dysfunction. The age-related degradation of the nuclear lamina affects the distribution of chromatin in the nucleus and leads to heterochromatin instability. At constitutively silenced, heterochromatin regions, loss of repressive H3K9me3 and DNA hypomethylation have been associated with spurious transcriptional activation of retrotransposons and neuroinflammation. Altered patterns of DNA methylation, histone marks and RNA methylation at regulatory elements and gene bodies of neuronal plasticity-related genes have been associated with impaired neuronal gene expression in the aged brain. Please note that the boxes “intact/impaired neuronal gene regulation” depict schematic summaries of age-related epigenetic changes reported for different neuronal genes in separate studies. The simultaneous profiling of the depicted epigenetic marks in the same neuron type has not yet been reported, and the depicted epigenetic patterns will likely differ depending on the specific genomic locus.

Epigenetic regulation takes place on multiple interconnected layers in the nucleus; from DNA methylation to histone modifications that influence chromatin compaction to the three-dimensional distribution of chromatin in the nucleus and post-transcriptional modifications (Willemin et al, 2024; Zocher and Toda, 2023). The coordinated interaction between these epigenetic layers is critical for the establishment and maintenance of neuron-specific gene expression patterns (Noack et al, 2022; Fernandez-Albert et al, 2019). For most of these epigenetic marks, substantial alterations have been described in the brain during the course of aging, which correlated with dysregulated gene expression in a locus-specific manner (Peleg et al, 2010; Benito et al, 2015; Zocher et al, 2021b; Zhang et al, 2022; Fig. 1). Indications for a functional role of epigenetic alterations in brain aging comes from manipulations of epigenomic regulator proteins, which profoundly affected neuronal gene regulation, behavior and cognitive abilities in young and aged mice (Oliveira et al, 2012; Benito et al, 2015; Lu et al, 2020a).

### Breakdown of neuronal DNA methylation landscapes

Neurons have unique DNA methylation landscapes which are highly specific for neuron type and brain region (Luo et al, 2017; Rizzardi et al, 2019; Clemens and Gabel, 2020). Single-cell DNA methylation sequencing has shown that even subtypes of interneurons and excitatory neurons as well as their spatial locations within the brain can be identified based on DNA methylation patterns (Luo et al, 2017; Liu et al, 2021; Yao et al, 2021), suggesting that DNA methylation contributes to the functional diversity of neurons. Neuronal DNA methylation landscapes are built up by DNA methyltransferases and recognized by methylation-sensitive transcription factors as well as DNA methylation reader proteins, such as Mecp2, that contain specific binding domains for methylated cytosines, to instruct neuronal gene expression (Kaluscha et al, 2022; Guy et al, 2011). Perturbations of DNA methylation enzymes and reader proteins have demonstrated that neurons are dependent on an active DNA methylation machinery for gene regulation, synaptic plasticity, learning, and memory (Feng et al, 2010; Morris et al, 2014; Gulmez Karaca et al, 2018; Yu et al, 2015; Kupke et al, 2024), and recent studies suggest that the functional maintenance of this machinery is impaired during aging (Oliveira et al, 2012; Zocher et al, 2021b).

DNA methylation patterns have been profiled during aging in a genome-wide manner in both rodent and human brains. Those studies found pronounced age-related alterations in DNA methylation in the hippocampus and prefrontal cortex, with locus-specific DNA hyper- or hypomethylation and a tendency toward reductions in global DNA methylation during aging (Numata et al, 2012; Masser et al, 2017; Hadad et al, 2018; Zocher et al, 2021b). DNA hypomethylation in the aged hippocampus has been shown to be particularly pronounced at enhancers and gene bodies of neuronal plasticity- and synapse-related genes, suggesting that the neuron-specific DNA methylation landscape is impaired in the aged brain (Zocher et al, 2021b). Yet, these findings were derived from sequencing of dissected brain tissues and should be confirmed in purified neuron populations in future experiments. The sources of those age-related alterations in DNA methylation still remain unknown and conflicting results have been reported regarding alterations in the levels of the enzymes of the DNA methylation machinery. While some studies have reported reduced expression of DNA methyltransferases or Tet DNA demethylases in the aged brain, other studies reported no age-related alterations in the levels of those enzymes in the brain (Chouliaras et al, 2011; Gontier et al, 2018; Hadad et al, 2018; Oliveira et al, 2012). A neuron type- and brain region-specific analysis of age-related changes in expression and genomic binding of DNA methylation enzymes is still missing but would give valuable insight into the sources of age-related DNA methylation changes in the brain.

Although age-related alterations of DNA methylation patterns have been extensively characterized, their functional consequences on neuronal gene expression programs and neuronal plasticity are still incompletely understood. Evidence for a role of DNA methylation as a driver of neuronal dysfunction in aging comes from its locus-dependent correlation with perturbed gene expression and from studies manipulating regulatory enzymes of DNA methylation in young and aged brains (Oliveira et al, 2012; Gontier et al, 2018). For instance, the DNA methyltransferase Dnmt3a is critical for hippocampal function in young animals and its overexpression in the aged hippocampus increased 5-methylcytosine levels and counteracted age-related cognitive decline (Oliveira et al, 2012). Moreover, overexpression of DNA demethylase Tet2 in the hippocampus of aged mice increased 5-hydroxymethylcytosine levels and promoted learning and memory (Gontier et al, 2018). In contrast, Tet2 overexpression in young mice impaired brain function (Pratt et al, 2022), presumably due to accelerated demethylation in young neurons. In addition, recent studies suggested that the activity of DNA demethylases Tet1 and Tet2 is required for reprogramming-induced rejuvenation of neuronal function in the aged retina (Lu et al, 2020a; for details, see “Neuronal reprogramming”), providing evidence that this functional improvement in aging is dependent on DNA methylation. Besides these broad perturbations of DNA methylation enzymes, there is little molecular insight into the consequences of DNA (de)methylation in aging. Particularly, how age-related alterations in DNA methylation affect neuronal gene regulation and neuronal plasticity is still incompletely understood. Our recent study suggested that hypomethylation of neuronal plasticity-related genes at genomic binding sites of Mecp2 correlated with impaired Mecp2 binding and aberrant transcriptional activation of those loci (Zocher et al, 2021b); thus providing a potential mechanism underlying neuron aging. Locus-specific manipulations of DNA methylation in young and aged neurons would help in future studies to decipher the functional consequences of DNA methylation in brain aging at the molecular, cellular and behavioral level.

### Altered histone marks and augmented chromatin accessibility

Post-translational modifications of histones, of which acetylation and methylation of histone lysine residues are the most characterized, critically contribute to neuronal epigenetic regulation by controlling the accessibility of chromatin to regulatory proteins such as transcription factors and RNA polymerases (Zhang et al, 2015). Single-cell profiling in the mouse brain showed that many histone modifications, such as H3K27ac or H3K27me3 which label active or silenced regulatory regions respectively, are distributed in a neuron type-specific manner and correlate with specific gene expression programs (Zhu et al, 2021). A few studies profiled alterations in histone modifications during neuron aging and related those to functional deficits in the aged brain. In general, these studies found decreases in both repressing histone marks at heterochromatin regions and activating histone marks at neuronal plasticity-related genes, with many of these effects being dependent on genomic locus, neuron type, and neuronal activation status (Peleg et al, 2010; Benito et al, 2015; Palomer et al, 2016; Zhang et al, 2022).

Histone methylation, such as H3K9me3 or H3K27me3, generally marks chromatin regions that are inaccessible and repressed in a context-dependent or constitutive manner (Zhang et al, 2015). Recent studies reported an age-related loss of repressive H3K9me3 in neurons, which correlated with increased chromatin accessibility and aberrant transcriptional activation of the affected loci (Zhang et al, 2022, 2023). For instance, Zhang and colleagues found that excitatory neurons in the hippocampus and prefrontal cortex lose H3K9me3 and gain chromatin accessibility at constitutively silenced, heterochromatin regions, such as LINE-1 repetitive elements, which was linked to the spurious transcriptional activation of those regions in aged neurons (Zhang et al, 2022). Chromatin accessibility was also increased at regions that contain binding sites of activity-induced transcription factors, such as Jun/AP-1/Atf3, suggesting that the binding of those transcription factors might be altered in aged neurons. Although the functional interaction between histone methylation, chromatin accessibility and gene expression during aging has not yet been addressed comprehensively, both increased accessibility and global reduction of constitutive heterochromatin likely result in neuronal gene dysregulation, particularly of genes that are normally silenced in unstimulated neurons. In addition, accumulation of repressive H3K9me3 and H3K27me3 at the promoters of neuronal plasticity genes during aging has been reported, which correlated with reduced transcript levels, suggesting that increased repressive histone methylation at specific neuronal genes might hamper neuronal plasticity during aging (Palomer et al, 2016). In line with this, acute pharmacological inhibition of histone lysine methyltransferase Suv39h1 before a learning and memory test promoted neuronal plasticity and cognitive abilities in aged mice (Snigdha et al, 2016). It is interesting that many of the age-related histone and chromatin accessibility changes have been found to be cell type-specific, with increased accessibility and reduced H3K9me3 in aged excitatory neurons but not in inhibitory neurons or glia (Zhang et al, 2022). Unraveling the consequences of neuron type-specific aging signatures would improve our understanding of neuronal epigenetic aging in the future.

Histone acetylation controls activity-dependent gene induction in neurons, and the enzymes that catalyze acetylation, histone acetyltransferases, play important roles in learning and memory (Alarcón et al, 2004; Wood et al, 2005; Oliveira et al, 2011; Malik et al, 2014). Histone deacetylases (HDACs), in contrast, typically negatively regulate cognitive processes (Fischer et al, 2007; Guan et al, 2009; McQuown et al, 2011), and the disturbed activity of these enzymes has been implicated in brain aging (Kwapis et al, 2018; Benito et al, 2015). Benito and colleagues found that, during aging, activating H4K12ac is reduced in excitatory neurons but not in non-neuronal cells of the dorsal hippocampus (Benito et al, 2015). This reduction was particularly pronounced at gene bodies of synaptic genes where it correlated with altered RNA splicing, specifically the aberrant inclusion of exons in aged neurons (Benito et al, 2015). In an earlier study, the same group reported that learning-induced accumulation of H4K12ac at activity-induced genes was impaired in the aged hippocampus, leading to disturbed induction of activity-induced gene expression programs in the aged brain (Peleg et al, 2010). Other studies found an age-related reduction of activating H3K27ac in the mouse and human brain (Palomer et al, 2016; Cheng et al, 2018). For example, reduced H3K27ac and increased H3K27me3 at the *Bdnf* promoter correlated with reduced transcript levels (Palomer et al, 2016). Rescue of age-associated changes in neuronal H4K12ac, H3K27ac and gene expression could be achieved by systemic treatment with HDAC inhibitor suberoylanilide hydroxamic acid, which also promoted cognitive abilities in aged mice (Peleg et al, 2010; Benito et al, 2015; Cheng et al, 2018). While these results suggest that accelerated deacetylation by HDACs underlies reduced histone acetylation in aged neurons, other studies reported that reduced H3K27ac in aged neurons also correlated with decreased binding of the histone acetyltransferase Cbp (Palomer et al, 2016). Likely, both the reduced maintenance of acetylation and accelerated deacetylation contribute to age-related alterations of the histone acetylation landscape.

### Disrupted nuclear architecture

In addition to chromatin accessibility at specific gene loci, neuronal epigenetic landscapes are shaped by nuclear architecture, i.e., the three-dimensional (3D) chromatin structure in the nucleus. Major advances in epigenomic sequencing technologies in the last years have made it possible to assess 3D chromatin structures of neurons in a genome-wide manner and even at single nucleus resolution (Liu et al, 2023; Tan et al, 2023; Fernandez-Albert et al, 2019). These studies found that neurons show characteristic 3D chromatin structures that differ between neuron types at specific neuronal genes, are activity-dependent and correlate with other epigenetic marks and gene expression (Liu et al, 2023; Fernandez-Albert et al, 2019). A few recent studies have suggested that the maintenance of the neuronal 3D chromatin architecture is impaired during aging (Tan et al, 2023; Zhang et al, 2022).

The distribution of chromatin in the nucleus is predominantly controlled by nuclear structural proteins, including nuclear lamins which anchor heterochromatin at the nuclear periphery. Age-related decreases in lamin protein levels have been reported in excitatory and inhibitory neurons in mouse, primate and human brains (Zhang et al, 2022, 2023), resulting in impaired nuclear integrity in aged neurons. For instance, Zhang and colleagues suggested that age-related reductions in lamin B1 and B2 levels in primate cortical neurons result in loss of heterochromatin and aberrant activation of repetitive elements, including endogenous retroviruses, ultimately inducing inflammatory pathways in aging neurons (Zhang et al, 2023). However, the consequences of age-related lamin reductions on genome-wide 3D chromatin distributions were not examined. Tan and colleagues profiled 3D chromatin distributions across the lifespan in the mouse and human cerebellum and found alterations in chromatin A/B compartments in cerebellar granule cells during aging (Tan et al, 2023). Specifically, they reported an increase in the fraction of ultra-long-range intrachromosomal contacts and a re-distribution of specific inter-chromosomal contacts in the aged mouse and human cerebellum. These data show that aging profoundly alters the nuclear architecture in neurons, yet the consequences of those alterations for neuronal epigenetic regulation and neuronal function remain to be investigated.

### Altered RNA metabolism

Neuronal function also depends on post-transcriptional mechanisms that control RNA stability, RNA degradation, RNA transport and translation, and particularly RNA modifications such as RNA methylation have been implicated as mediators of these processes (Livneh et al, 2020; Shi et al, 2018; Merkurjev et al, 2018). Recent studies have reported that RNA methylation patterns are disrupted in the aged brain, potentially contributing to cognitive decline (Castro-Hernández et al, 2023; Shafik et al, 2021). For instance, Castro-Hernández and colleagues profiled m^6^A RNA methylation in the hippocampus and cortex of 3-month-old and 16-month-old mice and found pronounced age-related hypomethylation at the gene bodies of synaptic plasticity-related RNAs, which they related to impairments in local translation at synapses (Castro-Hernández et al, 2023). Many of those RNAs were also hypomethylated in the cortex of Alzheimer’s disease patients, suggesting that aging- or neurodegeneration-related effects on RNA methylation are conserved in humans (Castro-Hernández et al, 2023). Shafik and colleagues also found age-related changes in RNA methylation in the mouse hippocampus and cortex, yet they reported a predominant hypermethylation of gene bodies in 1-year-old mice compared to 6-week-old adolescent mice (Shafik et al, 2021). A comprehensive characterization of purified neuron populations from multiple mouse ages might help to clarify in future experiments the dynamics of neuronal RNA methylation changes over the lifespan.

In addition to the classical epigenetic modifications described above, RNA molecules themselves can participate in transcriptional and post-transcriptional regulation, which is subject to age-related impairments. For instance, noncoding RNAs have emerged as important regulators of neuronal development and synaptic plasticity, and alterations in their levels have been linked to cognitive impairments (Schratt et al, 2006; Gao et al, 2010; Fischer, 2014). Transcriptomic profiling has shown that many noncoding RNAs, including lncRNAs and miRNAs, are dysregulated in the aged brain (Wagner et al, 2023; Hajdarovic et al, 2022; Danka Mohammed et al, 2016; Zovoilis et al, 2011; Butler et al, 2019; Ben-Tov Perry et al, 2023), with several of those having established functions as regulators of synaptic plasticity and cognition (Danka Mohammed et al, 2016; Danka Mohammed et al, 2017; Zovoilis et al, 2011; Butler et al, 2019; Ben-Tov Perry et al, 2023). The dysregulation of noncoding RNAs is likely connected to age-related alterations of classical epigenetic marks, such as DNA and histone modifications, although this interplay has not yet been comprehensively assessed during neuron aging. In other systems, it is known that the levels of noncoding RNAs are controlled by epigenetic marks (Gao et al, 2010; Glaich et al, 2019), and noncoding RNAs, in turn, can alter epigenetic modifications by guiding chromatin-remodeling proteins to regulatory genomic regions or through directly regulating the expression of epigenetic enzymes (Swahari et al, 2021; Butler et al, 2019; Khalil et al, 2009). It would be interesting to dissect, in future experiments, the functional interaction between noncoding RNAs and DNA/RNA/histone modifications during neuron aging.

Some non-canonical functions of RNA have also been linked to epigenetic regulation and might have roles in neuron aging. For instance, a recent study has demonstrated that RNAs in neuronal nuclei can be extremely stable, with some postnatally generated RNAs being retained over years in granule cells of the mouse hippocampus (Zocher et al, 2024). Those stable nuclear RNAs were suggested to be important for the long-term maintenance of heterochromatin and, thus, their decay could contribute to age-related impairments in neuronal epigenetic regulation. In addition, cytoplasmic RNA granules, localized assemblies of RNAs and their RNA-binding proteins, have been reported to be important for post-transcriptional regulation in neurons and be affected by aging (Bauer et al, 2023). RNA granules can be transported along neuronal processes and undergo stimulus-dependent de-condensation, which has been implicated in the activity-dependent translation of synaptic RNAs (Bauer et al, 2023). During brain aging in Drosophila, differences in RNA granule composition and progressively increased condensation into larger RNA granules have been observed, which is related to translational repression of the granule-associated RNAs (Pushpalatha et al, 2022). Yet the consequences of increased RNA granule condensation on neuronal function in aging are still unknown and should be clarified in future studies.

## Neuronal epigenomes—key targets of existing brain rejuvenation strategies?

**Figure 2:**
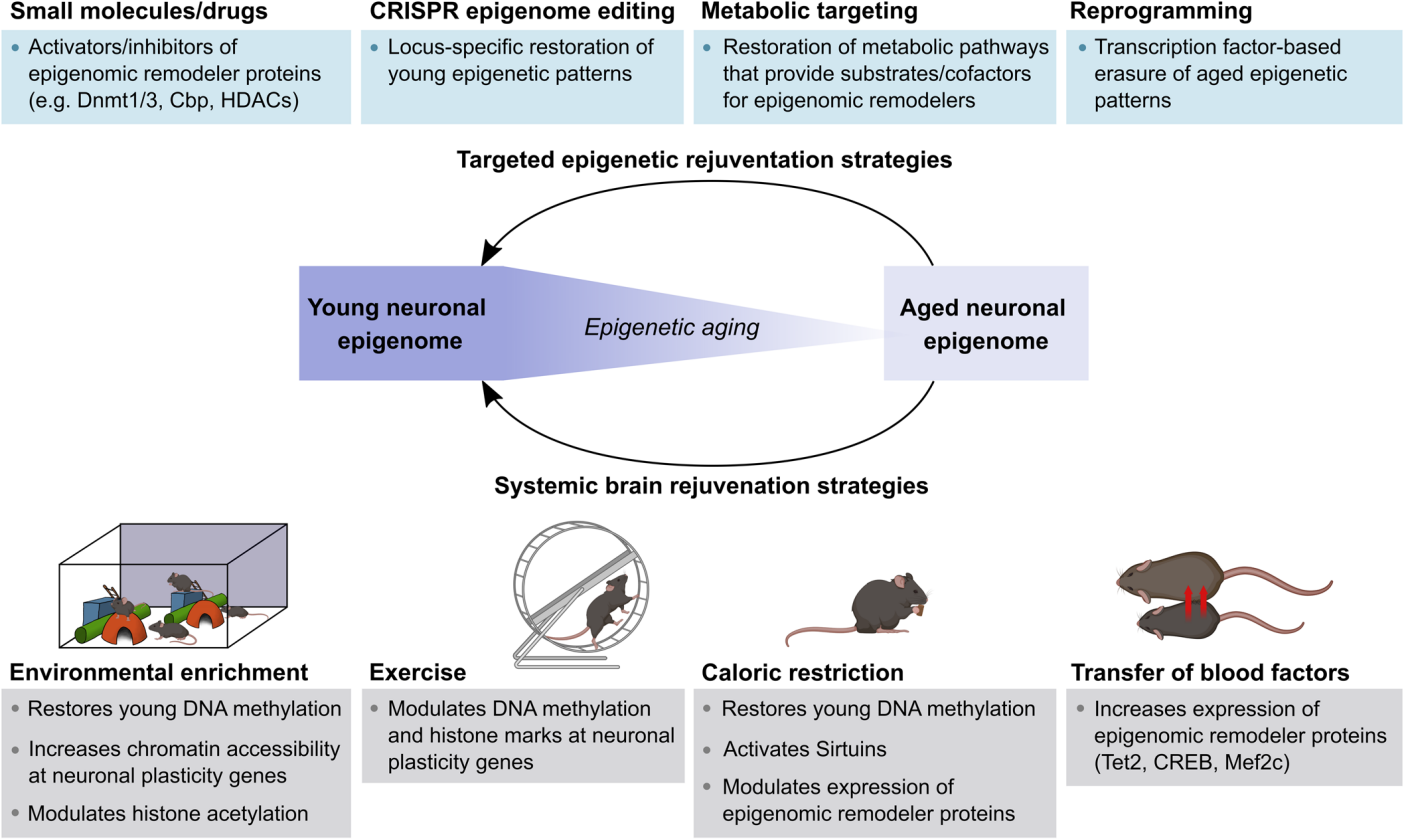
Epigenetic rejuvenation strategies to counteract cognitive decline. Summarized are potential strategies to specifically target neuronal epigenomes (top) and systemic brain rejuvenation interventions, for which modulating effects on the neuronal epigenome have been reported (bottom).

Neuronal epigenetic landscapes are remarkably malleable and plastic to environmental stimulation, and this epigenetic plasticity contributes to experience-dependent brain functions (Guo et al, 2011; Fernandez-Albert et al, 2019; Zocher et al, 2021b; Campbell and Wood, 2019). The malleability of the neuronal epigenome also means that its age-related alterations can potentially be prevented and/or reversed. In fact, recent studies have provided evidence that established brain rejuvenation strategies known to counteract age-related cognitive decline also slow down epigenetic aging (Zocher et al, 2021b; Hadad et al, 2018; Lu et al, 2020a; Zhang et al, 2020). These rejuvenation strategies have gained much attention in recent years and include, among others, lifestyle interventions, such as physical exercise, environmental enrichment, and caloric restriction, as well as the transfer of young blood-borne factors and cellular reprogramming (Benayoun et al, 2015; Zhang et al, 2020). However, it should be noted that because many of these interventions also stimulate brain function in young animals, it is still debated whether they specifically slow down aging processes (Keshavarz et al, 2023). Despite established improvements in brain function, mechanistic insight into how rejuvenation strategies affect neuronal function in the aged brain is still incomplete. This section summarizes recently accumulating evidence for the role of neuronal epigenetic plasticity as a functional mediator of selected brain rejuvenation strategies (Fig. 2).

### Environmental enrichment

Environmental enrichment refers to a lifestyle in which individuals receive environmental stimuli on multiple scales, including multisensory, physical, social, and cognitive stimulation. An enriched environment has been shown to improve brain function throughout the lifespan and to promote resilience against age-related cognitive decline and neurodegeneration (Nithianantharajah and Hannan, 2006; Kempermann, 2019). In rodent models of environmental enrichment, animals actively explore large cages, which is mediated by the intrinsic novelty-seeking behavior characteristic of rodents. Animal activity can be further enhanced by increasing social group size and through the frequent re-arrangement of toys, tunnels, and other objects that are placed in the cage, which provides repeated novelty exposure. In that way, enriched environments model an active lifestyle rich in experience, social exchange and physical activity, all of which is associated with healthy cognitive aging also in humans (Fratiglioni et al, 2004). Environmental enrichment has strong beneficial effects on neuronal function in aged brains—it promotes synaptic plasticity, dendritic outgrowth, spine formation as well as neuronal survival during adult hippocampal neurogenesis, among others (Van Praag et al, 2000). Due to the multifactorial nature of the stimulation, the molecular mechanisms underlying these effects in the brain have been difficult to characterize. Nevertheless, a few recent studies have suggested that epigenetic mechanisms are involved and that environmental enrichment can even slow down neuronal epigenetic aging.

Multiple studies have reported that environmental enrichment alters epigenetic modifications in the mouse brain, including DNA methylation (Irier et al, 2014; Zhang et al, 2018; Zocher et al, 2020, 2021b), histone acetylation (Fischer et al, 2007), chromatin accessibility (Barker et al, 2021; Espeso-Gil et al, 2021) and 3D chromatin architecture (Espeso-Gil et al, 2021; Box 1). In addition, previous studies suggested that environmental enrichment counteracts age-related DNA methylation changes in the hippocampus of mice (Zocher et al, 2021b; Penner et al, 2016). Specifically, we recently showed that the life-long housing of adult mice in an enriched environment prevented epigenetic aging at one-third of the age-sensitive cytosines (Zocher et al, 2021b). Even when environmental enrichment was started in old age, a youthful DNA methylation state was restored at many loci in the hippocampal dentate gyrus, providing evidence that age-related DNA methylation changes are indeed reversible in the brain. How environmental enrichment interferes with epigenetic aging in the brain is still unknown, but many of the environmentally sensitive loci overlapped neuronal activity-dependent epigenetic loci (Zocher et al, 2021b; Guo et al, 2011), suggesting that activity-mediated processes might be involved. The causal role of neuronal epigenomes in mediating the beneficial effects of environmental enrichment on brain function has not yet been addressed, and the functional consequences are still to be established. Nevertheless, in a previous study, environmental enrichment prevented age-related hypomethylation at binding sites of the methyl-CpG-binding protein Mecp2 and restored Mecp2 binding at synaptic plasticity-related genes in the aged hippocampus, which contributed to the environment-dependent regulation of those genes in the aged brain (Zocher et al, 2021b). Moreover, Barker and colleagues showed that environmental enrichment increased levels of the neuronal activity-regulated transcription factor Mef2c in neurons and increased chromatin accessibility and transcription of Mef2c target genes in the prefrontal cortex (Barker et al, 2021). They further showed that proper transcriptional regulation of synaptic plasticity-related genes and aspects of enriched environment-induced cognitive flexibility were dependent on Mef2 transcription factors in mice, suggesting a causal role of this epigenomic remodeling protein in mediating the benefits of environmental enrichment on the brain (Barker et al, 2021). Target genes of both factors, Mecp2 and Mef2c, were also enriched among the genes that predicted cognitive abilities in aged humans (Barker et al, 2021), suggesting a potential conservation of epigenomic mechanisms in mediating cognitive health in humans.

Box 1 Cell type-specificity of epigenetic aging and rejuvenation It should be noted that the majority of studies that have assessed epigenetic effects of rejuvenation strategies in the brain have analyzed epigenetic patterns in dissected hippocampus tissue and not selectively in neurons. Due to the predominant abundance of neurons in the hippocampus (Hochgerner et al, 2018; Methi et al, 2024), the detected epigenetic alterations likely originate from neurons, but a contribution of other cell types cannot be excluded. Besides neurons, also glial cells, including microglia, astrocytes, oligodendrocytes and neural stem cells, are affected by aging but stimulated by rejuvenating interventions, and their age-related impairments critically contribute to cognitive decline (Salas et al, 2020). Accumulating evidence suggests that epigenetic control is also important for glial cell function (Ayata et al, 2018; Lee et al, 2024) and age-related epigenetic and transcriptomic alterations have also been described in glial cells (Li et al, 2023; Cho et al, 2015; Habib et al, 2020; Yeo et al, 2023; Zocher and Toda, 2023). For instance, Li and colleagues have reported enhanced chromatin accessibility at the promoters of immune activation-associated genes in aged microglia, which correlated with enhanced transcription of those genes and might mediate the elevated neuroinflammation found in the aged brain (Li et al, 2023). Analyzing cell type-specific epigenetic aging signatures in the brain and the potential for their rejuvenation might aid our understanding of brain aging and cognitive decline in the future. Moreover, there is considerable communication between cell types in the brain, with neuronal function being controlled by signaling cues from glial cells. It would be interesting to investigate the influence of glial-derived signaling cues on the aging neuronal epigenome.

### Physical exercise

Physical exercise has well-known benefits on brain health in young and aged individuals—it enhances synaptic plasticity, hippocampal neurogenesis and cognitive abilities, and decreases the risk for neurodegenerative diseases (Cooper et al, 2018). The effects of physical activity on brain function are in part mediated by peripheral tissues, including liver, muscle, fat, heart and blood-resident cells, which secrete metabolites, peptides, extra-cellular vesicles and miRNAs with neuro-modulatory and potential epigenome-modulatory roles into the blood (Chow et al, 2022; Zhang et al, 2020). Epigenetic alterations in response to physical exercise have been described for several tissues (Kawamura et al, 2023; Murach et al, 2022) and recent studies suggest that also the neuronal epigenome is sensitive to physical exercise (Urdinguio et al, 2021).

Physical exercise altered the DNA methylation landscape in the hippocampus of young mice, with a predominant hypomethylation of synaptic plasticity-related genes (Urdinguio et al, 2021), similarly as observed after environmental enrichment (Zocher et al, 2021b). The exercise-induced DNA methylation changes were found at regulatory genomic regions, including binding sites of Mef2 transcription factors, and partially correlated with transcriptional alterations in the hippocampus (Urdinguio et al, 2021). Moreover, exercise altered patterns of H3K27me3 and H4K8ac in hippocampal neurons, which is related to neuronal gene expression changes (Raus et al, 2023). In addition, exercise has been suggested to modulate *Bdnf* expression in the hippocampus by reducing repressive H3K9me3 levels at one of its intragenic promoters (Ionescu-Tucker et al, 2021). Previous studies have also suggested that exercise influences the expression of epigenetic regulators in the brain (Jessop and Toledo-Rodriguez, 2018) and that exercise-induced hypomethylation is enriched at Tet1 binding sites in the genome (Urdinguio et al, 2021), suggesting that enhanced activation of the DNA (de)methylation machinery might underlie the epigenetic changes. Whether physical exercise in aged mice can reverse age-related epigenetic changes in neurons and whether epigenetic mechanisms are involved in mediating exercise-induced effects on neuronal function has, however, not yet been investigated.

### Caloric restriction

Caloric restriction has long been appreciated as an effective intervention in multiple species to extend lifespan and promote healthspan, including the ability to prevent and reverse age-related impairments in brain structure and function (Colman et al, 2009; Brandhorst et al, 2015). At the mechanistic level, caloric restriction targets nutrient-sensing pathways and results in metabolic reprogramming, reduced inflammation and enhanced autophagy, affecting multiple organs, including the brain (Mahmoudi et al, 2019). Accumulating evidence also suggests a contribution of epigenetic mechanisms in mediating the beneficial effects of caloric restriction on lifespan and brain function.

Several studies have demonstrated that caloric restriction protects from age-related epigenetic changes in multiple tissues. For instance, caloric restriction counteracted age-associated changes in DNA methylation in the hippocampus and altered the expression of DNA methyltransferases and Tet enzymes in the hippocampus (Chouliaras et al, 2011; Hadad et al, 2018). While caloric restriction has been shown to robustly reduce epigenetic age as measured using DNA methylation-based epigenetic clocks in liver (Hahn et al, 2017) and blood cells (Maegawa et al, 2017; Petkovich et al, 2017; Thompson et al, 2018), such analysis is still missing for the brain.

The life- and healthspan-extending effects of caloric restriction have been linked to the activity of sirtuins*—*a class of HDACs that is sensitive to cellular metabolic states (Satoh et al, 2017). Caloric restriction rescues the age-related reduction of Sirt1 expression in the hippocampus (Lardenoije et al, 2015), and brain-specific activation of Sirt1 is both sufficient and necessary for spine formation, synaptic plasticity, and cognitive abilities (Gao et al, 2010; Michán et al, 2010; Corpas et al, 2017). Sirtuins deacetylate histones and non-histone substrates and thereby promote silencing of specific genes. For instance, Sirt1 has been shown to promote hippocampal function by repressing brain-specific miR-134, leading to post-transcriptional activation of CREB (Gao et al, 2010). In addition to their gene-specific actions, sirtuins influence the global epigenetic landscape by interacting with other chromatin-remodeling enzymes. For instance, Sirt1 upregulates histone methyltransferase Suv39h1, thereby stimulating H3K9me3 deposition and promoting heterochromatin maintenance (Vaquero et al, 2007). Sirt1 also influences the DNA methylation machinery*—*it controls the activity of Mecp2 in the brain (Zocchi, 2012) and recruits Dnmt1, which is upregulated by glucose starvation (Li et al, 2010). In line with this, manipulation of Sirt1 expression influences DNA methylation at genomic targets of polycomb group proteins (Wakeling et al, 2015). However, the role of sirtuins in mediating the effects of caloric restriction on the neuronal epigenetic landscape still needs to be clarified. Taken together, there is evidence that epigenetic mechanisms are involved in the rejuvenating effects of caloric restriction on the brain, but mechanistic insight into how caloric restriction influences different epigenetic layers in aged neurons is still incomplete.

### Transfer of young blood factors

Blood-based strategies for brain rejuvenation have received much attention in recent years. Transfer of whole blood, plasma, purified cells or bioactive factors from young into aged mice reduces neuroinflammation, enhances synaptic plasticity, promotes hippocampal neurogenesis, and improves learning and memory in aged mice (Pluvinage and Wyss-Coray, 2020; Bieri et al, 2023). Both lack of pro-neuronal factors and accumulation of pro-inflammatory factors in aged blood, together with reduced selectivity of the blood-brain barrier in aging, have been reported as underlying mechanisms (Pluvinage and Wyss-Coray, 2020; Bieri et al, 2023). However, molecular changes in neurons that mediate the cognitive enhancement of blood transfer are incompletely understood. Suggestions of a role of epigenetic mechanisms come from a recent study which reported that blood exchange from young into aged mice increased the expression of DNA demethylase Tet2 in the aged hippocampus and that overexpression of Tet2 in the hippocampus improved cognition in aged mice (Gontier et al, 2018). However, those effects were predominantly related to increased hippocampal neurogenesis, and consequences on the neuronal DNA methylation landscape were not investigated. In a previous study, the same group showed that heterochronic parabiosis increased expression of chromatin-remodeling proteins such as Mef2c, Egr1, Klf6, and CREB in the aged hippocampus and that functional improvements after heterochronic parabiosis were dependent on phosphorylated CREB (Villeda et al, 2014). There is also evidence that the transfer of young blood factors rejuvenates DNA methylation patterns in liver and blood cells (Kawamura et al, 2023), suggesting epigenetic effects in peripheral organs. However, the effects of blood-based rejuvenation strategies on epigenomic aging signatures in the brain remain to be investigated.

### Neuronal reprogramming

The ectopic expression of pioneer transcription factors that reset cell type-specific epigenetic patterns has emerged as a potential epigenetic rejuvenation strategy in recent years (Cipriano et al, 2023). Initially recognized for their capacity to reprogram somatic cells to a pluripotent cell stage, the transcription factors Oct4, Sox2, Klf4, and Myc (together referred to as OSKM or the “Yamanaka factors”) have recently received attention for their ability to remove cellular and molecular features of aging, to promote disease resistance in aged mice and extend lifespan in models of premature aging (Ocampo et al, 2016). Cellular reprogramming is associated with major epigenetic remodeling which has been shown to be the mediator of the functional improvement in aging. OSKM induction erases epigenetic signatures of aging even in cells derived from very old subjects (Horvath, 2013; Petkovich et al, 2017). The potential of reprogramming as a brain rejuvenation strategy has just started to be explored, and there is no study yet reporting the use of reprogramming to counteract age-related cognitive decline in vivo. However, evidence suggests that reprogramming can restore neuronal function in aging and disease by targeting neuronal epigenomes (Lu et al, 2020a).

Li and colleagues reported that induction of Oct4, Sox2, and Klf4 (OSK) expression for a period of 4 weeks in retinal ganglion cells was sufficient to restore neuronal function and visual acuity of aged mice to the levels of young mice (Lu et al, 2020a). The functional improvement was associated with a reversal of age-related DNA methylation changes at synaptic plasticity genes and was dependent on the expression of Tet1 and Tet2, demonstrating that DNA demethylation mediates reprogramming-based neuronal rejuvenation. OSK induction further restored age-related transcriptional changes, with the vast majority of genes being downregulated by aging but upregulated by OSK (Lu et al, 2020a). Another study reported that cyclic OSKM induction increased H3K9me3 levels in hippocampal granule cells of 10-month-old mice and improved novel object recognition (Rodríguez-Matellán et al, 2020). Pharmacological inhibition of the H3K9me3 methyltransferase Suv39h1 abolished OSKM-induced rescue of molecular hallmarks of aging in peripheral tissues, such as increased H3K9me3, reduced DNA damage and altered nuclear lamina (Ocampo et al, 2016), further highlighting the dependence of reprogramming on epigenetic mechanisms. Although current research aims to clarify the therapeutic potential of reprogramming to treat age-related functional impairments, the results from reprogramming experiments substantiated the observation that age-related epigenetic changes are sources of tissue decline and can be targeted for functional neuron rejuvenation.

## Approaches for the specific manipulation of neuronal epigenomes

The epigenetic effects of the brain rejuvenation interventions described in the previous section support the notion that neuronal epigenomes are critical regulators for the maintenance of brain function in aging and can be targeted for functional rejuvenation. To erase aging signatures in neurons without global epigenetic perturbations, strategies for the specific manipulation of neuronal epigenomes are needed (Fig. 2). Those will help to further unravel the concrete functional implications of age-related epigenetic modifications and provide potential tools to restore youthful epigenetic states in neurons.

### Pharmacological modulation of epigenomic remodelers

Epigenetic patterns can be modulated by small molecules or drugs that alter the activity of enzymes of the epigenetic machinery. Several inhibitors of DNA methyltransferases, histone methyltransferases, histone acetyltransferases and HDACs have been identified and applied to the study of epigenetic regulation in the brain. For instance, pharmacological inhibition of DNA methyltransferases has helped to demonstrate the necessity of dynamic DNA methylation changes for gene expression, synaptic plasticity and learning and memory (Miller and Sweatt, 2007; Levenson et al, 2006). In the context of brain aging and neurodegeneration, HDAC inhibitors such as suberoylanilide hydroxamic acid have been shown to counteract age-related reductions in histone acetylation and improve brain function in aged mice and Alzheimer’s disease models (Fischer et al, 2007; Kilgore et al, 2010; Benito et al, 2015). Moreover, pharmacological activation of enzymes responsible for maintaining epigenetic patterns, such as DNA methyltransferases or histone methyltransferases and acetyltransferases, could potentially be used to preserve or restore neuronal epigenetic patterns in aging. However, while recent small-molecule screens identified methyltransferase inhibitors with lower toxicity and improved specificity for the specific enzymes (Huang et al, 2021; Sandoval et al, 2022; Newton et al, 2020; Pappalardi et al, 2021), only a few activators of epigenetic enzymes have yet been identified. A recent study showed that a small-molecule activator of histone acetyltransferases p300/Cbp promoted neuronal survival in a Parkinson’s disease model (Hegarty et al, 2016). Moreover, potential caloric restriction mimetics, such as rapamycin, nicotinamide and metformin, have been shown to alter epigenetic patterns, partially through the activation of sirtuins, yet their specific targets and efficacy remain to be investigated (Wu et al, 2018; Hao et al, 2022).

Taken together, the use of pharmacological interventions in neuronal epigenetic aging is currently limited by the availability of highly specific effectors with negligible cytotoxicity. Moreover, systemic administration of pharmacological modulators will affect the brain through epigenetic alterations in peripheral organs, which might restrict their use for studying specific epigenetic effects in brain aging as well as for therapeutic interventions.

### Locus-specific epigenomic editing

In recent years, tools for the locus-specific editing of epigenetic patterns based on the CRISPR/Cas9 system have been developed (Hilton et al, 2015; Kearns et al, 2015; Liu et al, 2016; Kwon et al, 2017; Liu and Jaenisch, 2019). These enable the specific manipulation of target genomic loci, such as regulatory regions that show epigenomic alterations during aging. Manipulation of epigenetic patterns is achieved by fusing the catalytic domains of epigenomic remodelers to catalytically inactive Cas9 (dCas9). By co-expressing these fusion dCas9-effector proteins with a cassette of sgRNAs, multiple loci can be simultaneously targeted. These systems have been described for the manipulation of DNA methylation (dCas9-Dnmt3a/-Tet1; Liu et al, 2016, 2018; Morita et al, 2016) and histone methylation or acetylation (dCas9-Suv39h1/-Ezh2/-Hdac1/-p300; Kearns et al, 2015; Hilton et al, 2015; O’Geen et al, 2017; Bohnsack et al, 2022). To increase the efficacy of epigenetic editing and achieve robust effects on gene expression, recent systems have employed the combinatorial recruitment of multiple epigenomic remodelers to one gene locus (Swain et al, 2024). These systems yielded efficient transcriptional repression simultaneously at multiple loci, albeit with high variation between loci (Swain et al, 2024).

CRISPR-based tools for epigenomic editing have been tested in multiple cell types, including neurons. For instance, Liu and colleagues used a dCas9-Tet1 fusion protein to demethylate an intragenic *Bdnf* promoter in primary neuron cultures, which resulted in pronounced upregulation of *Bdnf* expression and mimicked neuronal responses following depolarization (Liu et al, 2016). In a later study, the same team showed that targeted dCas9-Tet1-based demethylation of a CGG repeat region upstream of the *Fmr1* gene could rescue neuronal function in an in vitro model of Fragile X syndrome (Liu et al, 2018). More recently, Liu and colleagues combined dCas9-Tet1-based demethylation with locus-specific recruitment of CTCF using dCpf1-CTCF fusion proteins to reactivate Mecp2 expression from the inactivated X chromosome in neurons, which rescued the functional deficits of Rett syndrome in vitro (Qian et al, 2023). While these studies showed that neuronal epigenomes can be efficiently manipulated in vitro, only a few studies so far have performed locus-specific epigenome editing in vivo in the brain. In the mouse embryonic cortex, in vivo editing of DNA methylation and histone methylation was used to study epigenetic regulation during cortical neurogenesis and was achieved by electroporation-mediated delivery of dCas9-Tet1/dCas9-Ezh2 constructs (Albert et al, 2017; Noack et al, 2019). Delivery into adult brains requires virus-based methods and both adeno-associated viruses and lentiviruses have been used to target neurons in vivo with CRISPR/Cas9-based tools (Zheng et al, 2018; Liu et al, 2016; Swiech et al, 2015; Lu et al, 2020b; Park et al, 2022; Bohnsack et al, 2022). For instance, a recent study achieved pronounced hypermethylation of the *App* promoter in the mouse hippocampus after lentivirus-mediated delivery of dCas9-Dnmt3a and sgRNAs targeting *App* (Park et al, 2022). In vivo epigenomic editing of *App* reduced signs of Alzheimer’s pathology and rescued cognitive abilities in an *App* knock-in mouse model (Park et al, 2022). Together these studies showed that locus-specific epigenomic editing tools can be applied to manipulate neuronal epigenomes and restore neuronal function.

While epigenomic editing strategies have been applied to achieve functional improvements in neurological disease models, their application in the context of brain aging and age-related cognitive decline has not yet been reported. Locus-specific repair or mimicking of aging signatures in neuronal epigenomes would greatly facilitate our understanding of the causal role of epigenetic aging in neuronal gene regulation and cognitive decline. The therapeutic potential of CRISPR/dCas9-based epigenomic editing will, however, be dependent on overcoming current limitations, including potential off-target effects of dCas9 epigenomic effectors or sgRNAs, improved delivery methods and optimization of long-term persistence of epigenomic editing in the brain.

### Metabolic targeting of neuronal epigenomes

Metabolic dysregulation during aging is linked to cognitive decline and accumulating evidence suggests an intricate crosstalk between metabolism and epigenetic regulation (Haws et al, 2020; Brunet and Rando, 2017; Camandola and Mattson, 2017). Epigenetic enzymes are highly dependent on metabolites to act as their substrates or cofactors, and the levels of many of those metabolites can be modified pharmacologically or through dietary factors. Of particular interest in this context is the dependence of methylation marks on the availability of methyl donors (Bekdash, 2023). Previous studies reported impaired metabolic homeostasis in the aged brain, potentially impacting epigenetic regulation (Ding et al, 2021). Preserving metabolic homeostasis through targeting of intermediates or core enzymes is thus one approach that could restore epigenetic patterns in aged neurons. While mechanistic insight and functional links in metabolic-epigenetic crosstalk in aging are yet to be established, targeting neuronal epigenomes through metabolic interventions to prevent epigenetic aging represents a promising research avenue.

## Conclusions and perspectives

During aging, the neuronal epigenome undergoes pronounced alterations on multiple scales, with detrimental consequences for neuronal epigenetic regulation in the aged brain. The suggestion of a role for epigenetic aging as a driver of neuronal dysfunction comes from the locus-specific correlation of epigenetic changes with gene expression, from the evident effects of global perturbations of epigenomic remodeling enzymes on brain function, and from observations that systemic brain rejuvenation strategies also restore epigenetic patterns in aged brains. To provide further evidence for a causal role of epigenetic aging in cognitive decline, locus-specific epigenetic editing of aging signatures in young and aged neurons will be required, which will also help to address the yet unknown functional interaction between different epigenetic layers during aging (Box 2). Once mechanistic insight is established, targeting neuronal epigenomes to prevent or repair epigenetic aging signatures could provide potential means to counteract brain aging and cognitive decline. Understanding how established brain rejuvenation strategies, such as environmental enrichment or caloric restriction, restore neuronal epigenomes in aging, will help to identify systemic modifiers of epigenetic aging. Moreover, intensifying research into the causes of neuronal epigenetic aging will help to develop strategies to prevent epigenetic aging and maintain brain function over the lifespan.

Box 2 Questions and future directions Neuronal epigenomes are critical determinants of neuronal identities and functions. Aging profoundly alters the neuronal epigenome, yet these epigenetic aging signatures are malleable. To understand the contribution of epigenetic aging to age-related cognitive decline and the potential of rejuvenating interventions, a number of key questions need to be answered:

1. How does neuronal epigenetic aging affect gene regulation and neuronal function?
2. Is there a functional interaction between different epigenetic layers during aging? Are some epigenetic marks more age-sensitive than others?
3. What causes epigenetic aging in neurons?
4. Are epigenetic aging signatures neuron type- and brain region-specific? Is there a selective vulnerability of specific neuron populations to epigenetic aging?
5. Does neuronal epigenetic plasticity mediate the beneficial effects of systemic brain rejuvenation strategies?
6. Can targeted interventions prevent epigenetic aging or restore a youthful epigenome long-term to preserve brain function in old age?

## Data Availability

The source data of this paper are collected in the following database record: biostudies:S-SCDT-10_1038-S44318-024-00148-8.
